# Impact of fatigue in Crohn's disease is negatively related to resting state functional connectivity between the superior parietal lobule and parahippocampal gyrus/hippocampus

**DOI:** 10.3389/fnhum.2025.1561421

**Published:** 2025-04-25

**Authors:** Theresa A. McIver, Charles N. Bernstein, Ruth Ann Marrie, Chase R. Figley, Md Nasir Uddin, John D. Fisk, Lesley A. Graff, Ronak Patel, Erin L. Mazerolle, Jennifer Kornelsen

**Affiliations:** ^1^Department of Radiology, Max Rady College of Medicine, Rady Faculty of Health Sciences, University of Manitoba, Winnipeg, MB, Canada; ^2^IBD Clinical and Research Centre, University of Manitoba, Winnipeg, MB, Canada; ^3^Department of Internal Medicine, Max Rady College of Medicine, Rady Faculty of Health Sciences, University of Manitoba, Winnipeg, MB, Canada; ^4^Department of Medicine, Dalhousie University, Halifax, NS, Canada; ^5^Neuroscience Research Program, Kleysen Institute for Advanced Medicine, Winnipeg Health Sciences Centre, Winnipeg, MB, Canada; ^6^Department of Neurology, School of Medicine and Dentistry, University of Rochester, Rochester, NY, United States; ^7^Department of Biomedical Engineering, Hajim School of Engineering and Applied Sciences, University of Rochester, Rochester, NY, United States; ^8^Nova Scotia Health and Departments of Psychiatry and Medicine, Dalhousie University, Halifax, NS, Canada; ^9^Department of Clinical Health Psychology, Max Rady College of Medicine, Rady Faculty of Health Sciences, University of Manitoba, Winnipeg, MB, Canada; ^10^Department of Psychology, Computer Science, and Biology, St. Francis Xavier University, Antigonish, NS, Canada

**Keywords:** Crohn's disease, functional connectivity, disease activity, fatigue, fMRI

## Abstract

**Introduction:**

Crohn's disease is one phenotype of inflammatory bowel disease (IBD). Fatigue is a common and burdensome symptom for persons with Crohn's disease. Despite its detrimental impact on health-related quality of life, the pathophysiology of fatigue in Crohn's disease is not fully understood. Specifically, basic research on the difference in brain functioning associated with fatigue in Crohn's disease is scarce. This study aimed to address this knowledge gap by identifying fatigue-related differences in brain resting state functional connectivity. in Crohn's disease.

**Methods:**

Participants included 49 adults with Crohn's Disease (*M*_age_ 53 yrs, 35 females) and 49 healthy controls (*M*_age_ 50 yrs, 31 females). The Fatigue Impact Scale (FIS) assessed impact of fatigue across three domains (physical, cognitive, and psychosocial) as well as total impact of fatigue. The Harvey-Bradshaw Inventory (HBI) assessed disease activity. Magnetic Resonance Imaging of brain functional connectivity during resting state (i.e.,: wakeful rest) was assessed in relation to scores on the FIS (total and for each domain). Moderation analyses tested whether brain resting state functional connectivity moderates the relationship between disease activity and fatigue.

**Results:**

The Crohn's disease group reported more severe fatigue than the healthy control group in each domain of the FIS. For the Crohn's disease group, increasing fatigue was associated with decreasing synchronicity of brain function (i.e., functional connectivity) between the superior parietal lobule and the parahippocampal gyrus/hippocampus. Unlike in the healthy control group, an increasing impact of physical fatigue was associated with decreasing functional connectivity between these ROIs for the Crohn's disease group (TFCE = 16.88, p-FDR = 0.03). Moderation analyses revealed a significant interaction between disease activity, total fatigue, and functional connectivity of the right superior parietal lobule and left anterior parahippocampal gyrus (ΔR^2^ = 0.058, F = 5.445, *p* = 0.0245). Higher scores on the HBI were only associated with higher total scores on the FIS in persons with Crohn's disease who exhibited negative functional connectivity between these brain regions.

**Discussion:**

In people with Crohn's disease, fatigue increases as functional connectivity between brain regions involved in sensorimotor integration and memory processing decreases.

## 1 Introduction

Crohn's disease is an immune-mediated inflammatory condition included in inflammatory bowel disease (IBD) (Singh and Bernstein, 2022). In addition to the gastrointestinal symptoms that arise from chronic, alternating periods of remitting and relapsing inflammation of the gastrointestinal tract (Burisch and Munkholm, 2015), fatigue is common (Vogelaar et al., 2013). Although estimates vary, fatigue affects roughly 50% of people with IBD (Nocerino et al., 2019); with approximately 72% of those experiencing an active episode reporting the burdensome symptom (Graff et al., 2011; D'Silva et al., 2022). Fatigue has also been reported at greater prevalence among persons with Crohn's disease than among those with ulcerative colitis (D'Silva et al., 2022; Grimstad et al., 2015; Regueiro et al., 2022), with its negative impact on health-related quality of life positioning it as a top-rated research priority (Radford et al., 2020; Dibley et al., 2016; Cohen et al., 2014). Despite its prevalence and detrimental impact on quality of life (Gibble et al., 2024), the pathophysiology of fatigue in Crohn's disease is not yet fully understood. While a variety of risk factors for fatigue have been established (Artom et al., 2016; Villoria et al., 2017; Schreiner et al., 2020; Pal et al., 2023), recent research has found that only 49% of fatigue is explained by composite factors representing disease activity, chronicity, and nutritional deficits (Christensen et al., 2022). This gap in our understanding of the mechanisms underlying fatigue in Crohn's disease limits opportunities to target interventions to manage this common symptom. A better understanding of the pathophysiology of fatigue in Crohn's disease may be achieved by examining the functional neural correlates representing changes in the brain-gut axis of those with Crohn's disease.

The brain-gut axis is a bidirectional communication system between the autonomic, enteric, and central nervous systems and the hypothalamic-pituitary-adrenal axis (Carabotti et al., 2015). The brain-gut axis monitors and integrates gut functions, and links emotional and cognitive centers of the brain with peripheral intestinal functions (Carabotti et al., 2015). Advances in our understanding of the brain-gut axis have been supported by human brain magnetic resonance imaging (MRI) research exploring neural biomarkers of IBD. Structural and functional neuroimaging techniques have identified IBD-related differences in brain morphology as well as resting state function (Yeung, 2020; Kong et al., 2020).

Neuroimaging research in IBD has also begun to explore the neural correlates of common symptoms external to the gut, including fatigue. The limited MRI research exploring neural correlates of fatigue in IBD has focused on Crohn's disease. Thapaliya et al. (2022) showed that participants with Crohn's disease exhibited an inverse relationship between fatigue and gray matter volume in the right supplementary motor area and white matter volume in the left cerebellum, as well as cortical thickness of the right parahippocampal gyrus, frontal pole, left temporal fusiform gyrus, orbitofrontal cortex, inferior temporal gyrus, postcentral gyrus, and middle frontal gyrus. However, when this same group subsequently explored the relationship between fatigue and resting state functional connectivity in Crohn's disease, they found no significant association (Thapaliya et al., 2023). Comparing resting state fMRI between participants with Crohn's disease and ulcerative colitis, as well as healthy controls, Thomann et al. (2020) included fatigue as a variable of interest in a joint-independent component analysis (ICA) assessing cross information from voxel-based morphometry (VBM) and resting state activity but also found no significant association for fatigue. More recently, the same group assessed gray matter volume differences related to fatigue in Crohn's disease, relative to active disease or remission (Thomann et al., 2024). After identifying brain regions that significantly differed in gray matter volume between those in active disease or remission, they examined correlations between gray matter volume and fatigue (Thomann et al., 2024). They identified the left precentral gyrus and right gyrus rectus as having a negative association between gray matter volume and fatigue for those in remission (Thomann et al., 2024). An additional exploratory whole-brain analysis of correlations between gray matter volume and fatigue revealed a positive association for the right middle temporal gyrus and cerebellum, as well as the left inferior temporal gyrus (Thomann et al., 2024). Despite such findings of fatigue-related structural differences, the relationship between fatigue and resting state functional connectivity in Crohn's disease has yet to be elucidated.

Since fatigue can impact different domains of functioning, a more comprehensive understanding of related neural mechanisms may be achieved by testing the association between functional connectivity and the domain-specific impact of fatigue (e.g., physical, cognitive, and psychosocial). Finally, despite being closely linked, fatigue in IBD is not exclusively predicted by disease activity, highlighting an opportunity to explore potential moderating factors of that relationship. Given that previous research has only revealed fatigue-related structural differences in Crohn's disease, the present study focuses on characterizing the functional neural correlates of fatigue in Crohn's disease. Specifically, the present study aims to: (1) Explore how the self-reported impact of fatigue on physical, cognitive, and psychosocial functioning is related to brain resting state functional connectivity among those with Crohn's disease; (2) Examine the interaction between disease activity, brain resting state functional connectivity, and the impact of fatigue in Crohn's disease.

Given that elevated fatigue in IBD is a well-established extraintestinal symptom, we expect the self-reported impact of fatigue to be elevated in Crohn's disease compared to healthy control participants. While the association between fatigue and brain function in IBD has yet to be elucidated, fatigue has repeatedly been linked to functional hypoconnectivity in chronic fatigue syndrome (Boissoneault et al., 2016; Zinn et al., 2016). We therefore hypothesize that within Crohn's disease, increasing fatigue will be associated with decreasing functional connectivity, and that the association between fatigue and resting state functional connectivity will differ between those with Crohn's disease and healthy controls. We also hypothesize functional connectivity associations with the impact of fatigue may vary relative to physical, cognitive, and psychosocial function. Finally, since fatigue is a complex symptom that is influenced by multiple factors, we hypothesize that the positive relationship between disease activity and fatigue in Crohn's disease may be moderated by fatigue-related functional connectivity.

## 2 Methods

### 2.1 Participants

Studies were approved by the University of Manitoba Health Research Ethics Board and all participants provided informed consent. Adults with Crohn's disease were recruited through a parent longitudinal study investigating psychiatric comorbidity in immune-mediated inflammatory disease (IMID study) (Marrie et al., 2018) and adult healthy controls were recruited through a separate sub-study (Uddin et al., 2022). Inclusion and exclusion criteria as per the parent longitudinal study are provided in Supplementary material and have previously been published (Boissoneault et al., 2016; Zinn et al., 2016). Only participants who underwent MRI were included in the present sub-study, resulting in a sample of 49 participants with Crohn's disease and 49 healthy controls.

### 2.2 Measures

All participants reported their date of birth and sex. For participants with Crohn's disease, disease duration and phenotyping based on the Montreal classification system (Satsangi, 2006) were recorded (Table 1). Disease severity/activity was assessed by trained staff using the Harvey-Bradshaw Inventory (HBI) (Evertsz et al., 2013) for those with Crohn's disease (Table 1), with active disease defined as a score of 5 or higher.

**Table 1:** Demographics, disease metrics, self-report measures.

	**CD (*n =* 49)**	**HC (*n =* 49)**	**Test statistic**
Age, years	52.85 ± 13.85 (22–77)	47.56 ±14.28 (19–81)	*t*_(96)_ = 1.86, *p =* 0.066
Sex, women	35 (71%)	30 (61%)	χ2_(1)_ = 1.142, *p =* 0.285
Active (HBI ≥5)/Inactive disease	16 (35%)/30(65%)^*^		
CD Location	20 L1 (41%); 5 L2 (10%); 24 L3 (49%); 1 L4 (2%)		
CD Behavior	17 B1 (35%); 17 B2 (35%); 18 B3 (37%); 5 P (10%)		
FIS_Physical	10.63 ± 10.01 (0–40)	2.84 ± 4.40 (0–18)	*t*_(65.87)_ = 4.99, *p < * 0.001
FIS_Cognitive	11.18 ± 9.93 (0–38)	3.96 ± 4.99 (0–20)	*t*_(70.76)_ = 4.55, *p < * 0.001
FIS_Psychosocial	18.57 ± 18.38 (0–71)	4.53 ± 6.58 (0–27)	*t*_(60.10)_ = 5.04, *p < * 0.001
FIS_Total	40.39 ± 37.0 (0–149)	11.33 ± 14.99 (0–57)	*t*_(63.35)_ = 5.10, *p < * 0.001
HADS-D	3.96 ± 3.77 (0–14)	1.22 ± 1.56 (0–7)	*t*_(63.90)_ = 4.69, *p < * 0.001

Age, HADS-D, and FIS (total and subscales) reported in mean ± standard deviation (range). Sex and disease activity reported by number of participants, with percentage of participants who were women and the percentage of those in active vs. inactive disease state provided in brackets. HC, healthy controls; CD, Crohn's disease; L1, ileum; L2, colon; L3, small bowel/colon; L4, upper GI which can accompany any of the L1, L2, or L3; B1, inflammatory; B2, fibrostenosing; B3, fistula; P, perianal disease; HADS-D, Hospital Anxiety and Depression Scale- Depression subscale; FIS, Fatigue Impact Scale. ^*^Data are missing for three CD Harvey-Bradshaw scores.

All participants completed the Fatigue Impact Scale (FIS) (Fisk et al., 1994; Marrie et al., 2023). This validated self-report measure consists of 40 items that assess the impact of fatigue across three domains: Cognitive functioning (10 items); Physical functioning (10 items); and Psychosocial functioning (20 items). Participants were asked to rate the extent to which fatigue has caused problems for them (0 = “no problem” to 4 = “extreme problem”) in relation to exemplar statements (e.g., “I am less able to complete tasks that require physical effort”). Symptoms of depression were assessed for all participants using the Hospital Anxiety and Depression Scale (HADS) (Zigmond and Snaith, 1983). The HADS is a validated measure commonly used for medical patients. Seven items assess depression, each scored from 0 to 3 and summed to a total, with clinically meaningful symptom elevation assumed for scores >11.

### 2.3 MRI data acquisition

As detailed in Uddin et al. (2022), anatomical and functional neuroimaging data were acquired using a 3T Siemens TIM Trio MRI system and a Siemens 32-channel receive-only head coil (Siemens Healthcare, Erlangen, Germany). High resolution anatomical, whole-brain T1-weighted (T1w) images were acquired using a 3D magnetization prepared rapid acquisition gradient-echo (MPRAGE) sequence and resting state fMRI data were acquired using a gradient-echo MB-EPI sequence. All scanning parameters are provided in Supplementary material and described in the study protocol publication detailing the longitudinal parent study under which the present data were collected (Uddin et al., 2022).

### 2.4 Analysis

#### 2.4.1 Self-report measures and clinical presentation

Analyses on self-report and clinical data were completed in SPSS (version 28.0.0.0). Participant characteristics were summarized using descriptive statistics. Bivariate comparisons used *t-*tests and chi-square tests as appropriate (Table 1).

#### 2.4.2 Neuroimaging data: pre-processing and analysis

Preprocessing and analyses were completed in CONN (Whitfield-Gabrieli and Nieto-Castanon, 2012) software [release 18.b (Nieto-Castanon, 2018)] and SPM (Penny et al., 2011) (release 12.7771). Functional and anatomical data were preprocessed using a flexible preprocessing pipeline (Nieto-Castanon, 2020a) including removal of initial scans, realignment with correction of susceptibility distortion interactions, outlier detection, direct segmentation and MNI-space normalization, and smoothing. Full details on preprocessing and denoising are described in Supplementary material.

First-level analyses proceeded with the calculation of Fisher-transformed bivariate correlation coefficients between all region-of-interest (ROI) pairs in a 132 ROI matrix. The 132 ROIs consisted of Harvard-Oxford atlas parcellations (Desikan et al., 2006) that fully spanned the cortical, subcortical, and cerebellar aspects of the brain. The correlation coefficients between every possible pair of the 132 ROIs were calculated using a General Linear Model [GLM; correlation (bivariate) setting with no weighting applied (Nieto-Castanon, 2020b)] for each participant. These values were then used in second (group)-level regression analyses to estimate correlation coefficients.

The primary functional connectivity analyses aimed to explore the relationship between functional connectivity of each ROI pair in the 132 ROI matrix and the impact of fatigue in each domain for people with Crohn's disease (i.e., main effect of fatigue, within each domain, in persons with Crohn's disease). Second (group)-level GLM regression analyses were performed separately using scores on the FIS overall (FIS_Total) and the subscales (FIS_Physical, FIS_Cognitive, and FIS_Psychosocial) as the covariate of interest and controlling for age and sex. Follow-up analyses aimed to confirm that the Crohn's disease group differed from healthy controls in the association between fatigue and functional connectivity from the ROI pairs identified in the primary exploratory analyses. All ROI-pairs that exhibited functional connectivity correlated with fatigue for the Crohn's disease group were used to generate the restricted ROI-matrix for the Crohn's disease vs. healthy control contrasts. To that end, the restricted matrix tested 15 connections among six ROIs [right and left superior Parietal Lobules (SPL), right anterior supramarginal gyrus (aMSG), left hippocampus (Hipp), and left anterior and posterior parahippocampal gyri (PHG)]. Separate Crohn's disease vs. healthy control contrasts were run for scores on the FIS_Total, FIS_Physical, and FIS_Cognitive. No Crohn's disease vs. healthy control contrast was completed for the FIS_Psychosocial given the null results for scores in that domain for the Crohn's disease and healthy control groups separately. For all second (group)-level analyses, connection-level hypotheses were evaluated using multivariate parametric statistics with random-effects across participants and sample covariance estimation across multiple measurements. Inferences were performed at the level of individual clusters (groups of contiguous connections). Cluster-level inferences were based on non-parametric statistics using Threshold Free Cluster Enhancement (TFCE) (Smith and Nichols, 2009) with 1,000 residual-randomization iterations. Results were thresholded using a familywise corrected p-FWE < 0.05 TFCE-score threshold, while controlling for age and sex.

#### 2.4.3 Moderation analyses

Our primary functional connectivity analysis identified pairs of brain regions between which resting state functional connectivity was related to fatigue in persons with Crohn's disease. The possible interaction between brain functional connectivity, disease activity, and fatigue was explored via moderation analyses based on these results of the initial group-level, whole-brain, ROI analyses of the Crohn's disease group. To test the hypothesis that the positive relationship between disease activity and fatigue would be moderated by resting state functional connectivity in Crohn's disease (Supplementary Figure 2), five moderation analyses were performed, with the moderator in each model being the functional connectivity between one of the following ROI-pairs from the Crohn's disease group: Model (1) right SPL and left anterior Parahippocampal Gyrus (aPHG); Model (2) right SPL and left posterior Parahippocampal Gyrus (pPHG); Model (3) right SPL and left Hipp; Model (4) left SPL and left aPHG; Model (5) left SPL and left pPHG. Moderation analyses were performed using the PROCESS macro (Hayes, 2018) within SPSS. Using an ordinary least squares path analysis approach, conditional effects were calculated to perform simple slope analyses at low (16th percentile), medium (50th percentile), and high (84th percentile) levels. These analyses included a bias correction with 5,000 bootstrapped samples and confidence intervals of 95%.

## 3 Results

### 3.1 Measures

Descriptive and inferential statistics for demographic and clinical characteristics are summarized in Table 1. Those with Crohn's disease reported significantly higher fatigue than the healthy control group across every domain, as well as higher scores for depressive symptoms according to the HADS-D (Table 1). The groups did not differ by sex or age. Crohn's disease medication use is detailed in Supplementary material.

### 3.2 Functional connectivity

**Figure 1:**
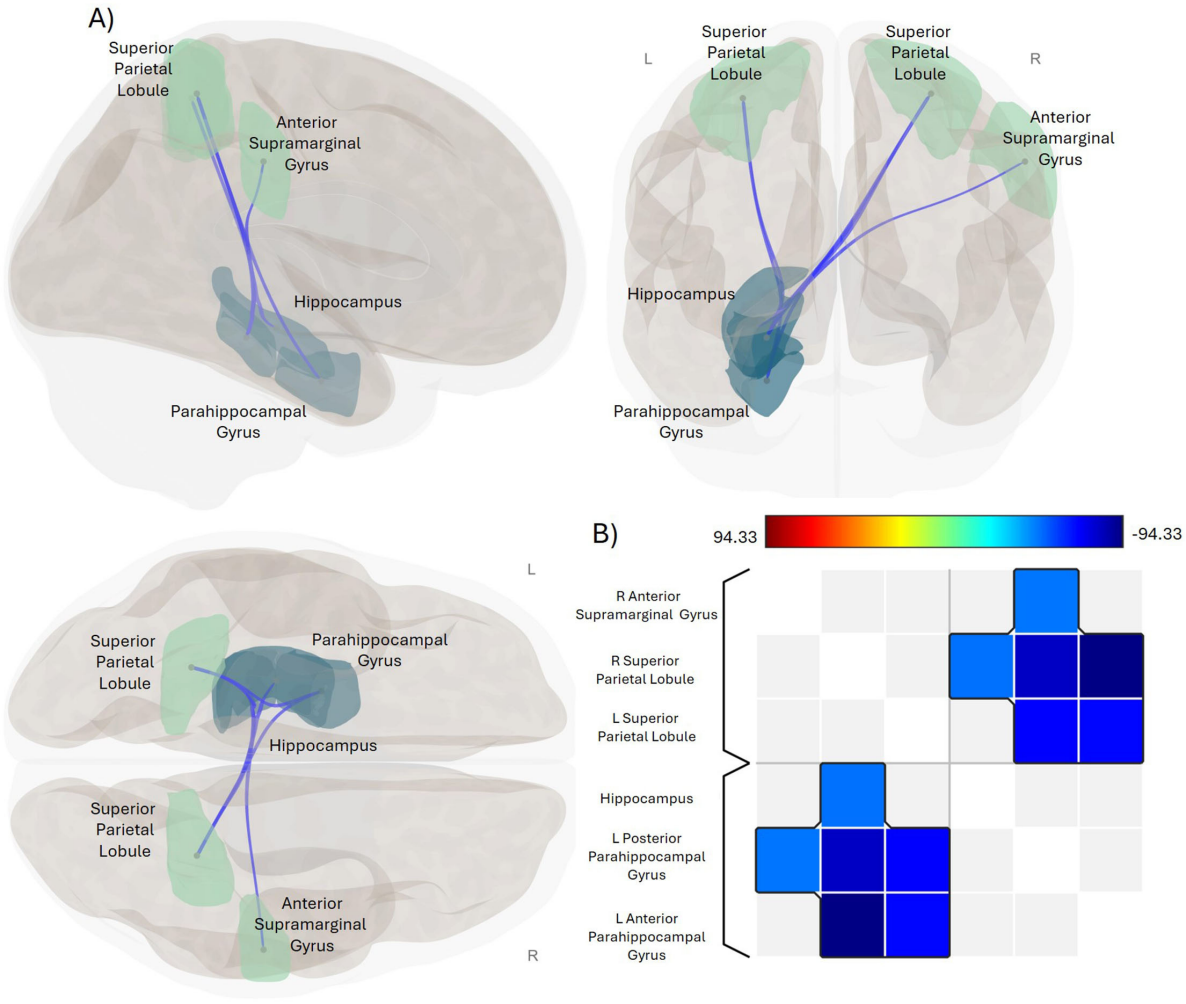
Significant negative association between impact of fatigue and functional connectivity in CD. **(A)** Glass brain display of the ROI-pairings that exhibit a statistically significant association between functional connectivity and the impact of fatigue in the physical domain for the CD group. Sagittal view is displayed in the upper left, coronal in the upper right, and axial in the lower left positions. Green-colored ROIs are parietal lobe regions, navy-colored ROIs are temporal lobe regions. Blue lines indicate significant negative association between functional connectivity of these ROI-pairings and FIS_Physical scores. Results were thresholded using a familywise corrected p-FWE < 0.05 TFCE-score threshold. R, right; L, left. **(B)** Heat map visualization matrix for the correlations between FIS_Physical scores and the functional connectivity of these ROI-pairings. Color bar shows range for TFCE-score test statistic.

The independent group-level regression analyses exploring the main effect of fatigue in the Crohn's disease and healthy control groups separately revealed that the impact of fatigue was negatively related to functional connectivity in the Crohn's disease group for FIS_Physical and FIS_Cognitive scores, as well as for the FIS_Total (Table 2, Figure 1). Specifically, scores on the FIS_Physical, FIS_Cognitive, and FIS_Total were negatively related to functional connectivity between the bilateral SPL and the left PHG (anterior and posterior divisions), as well as between the right SPL and left Hipp (Table 2, Supplementary Figure 1). In addition, scores on the FIS_Physical were negatively related to functional connectivity between the anterior division of the right aSMG (Table 2, Figure 1). The impact of fatigue on the psychosocial domain of functioning (FIS_Psychosocial) was not related to functional connectivity for the Crohn's disease group. Independent group-level regression analyses for the healthy control group did not reveal any relationship between functional connectivity and the FIS_Total or within any separate domain of functioning.

**Table 2:** Results of the group- level, whole-brain, ROI-to-ROI analysis testing relationship between functional connectivity and the impact of fatigue, separated by group (CD; HC).

**Group**	**Significant connections**	**Test statistic**	**Threshold free cluster enhancement FDR-corr *p* value**	**Analysis-level, ROI-to-ROI connection uncorrected *p* value**
**CD**				
*FIS_Physical*	Cluster 1:	*TFCE* = 94.33	0.017	–
	right SPL – left aPHG	*t*_(45)_ = −4.78		0.000019
	right SPL – left pPHG	*t*_(45)_ = −4.21		0.000012
	left SPL – left pPHG	*t*_(45)_ = −3.72		0.00056
	left SPL – left aPHG	*t*_(45)_ = −3.67		0.00063
	right SPL – left Hipp	*t*_(45)_ = −2.67		0.01
	right aSMG – left pPHG	*t*_(45)_ = −2.59		0.012
*FIS_Cognitive*	Cluster 1:	*TFCE* = 78.11	0.044	–
	right SPL – left aPHG	*t*_(45)_ = −4.51		4.6E-05
	left SPL – left aPHG	*t*_(45)_ = −4.29		9.4E-05
	left SPL – left pPHG	*t*_(45)_ = −3.89		0.00033
	right SPL – left pPHG	*t*_(45)_ = −3.76		0.00049
	right SPL – left Hipp	*t*_(45)_ = −2.56		0.014
*FIS_Psychosocial*	–	–	–	–
*FIS_Total*	Cluster 1:	*TFCE* = 93.33	0.01	–
	right SPL – left aPHG	*t*_(45)_ = −4.92		1.2E-05
	left SPL – left aPHG	*t*_(45)_ = −4.10		0.00017
	right SPL – left pPHG	*t*_(45)_ = −4.00		0.00023
	left SPL – left pPHG	*t*_(45)_ = −3.67		0.00064
	right SPL – left Hipp	*t*_(45)_ = −2.59		0.013
HC	–			

**Figure 2:**
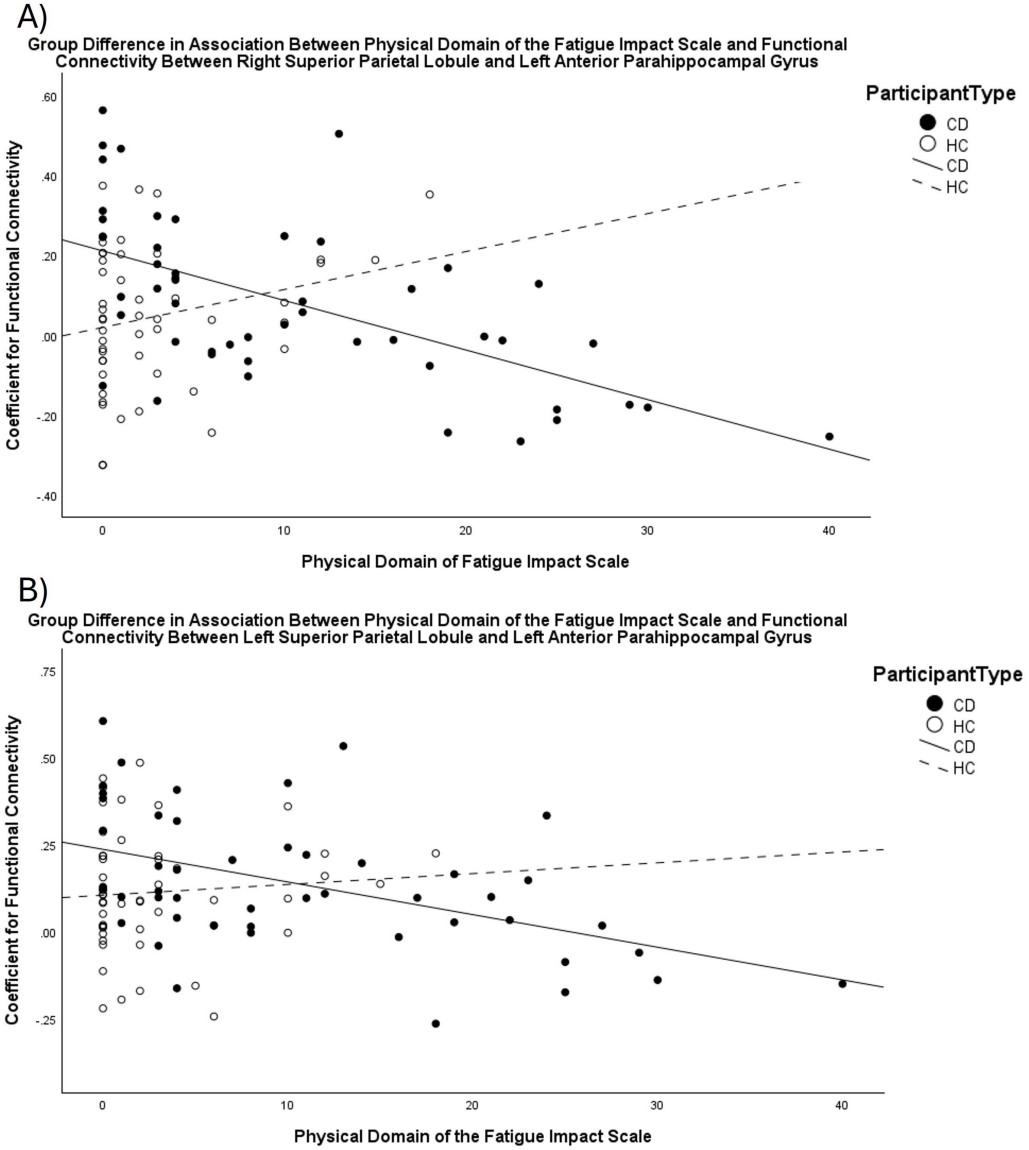
Scatterplots displaying the different relationship between scores on the FIS_Physical and functional connectivity for CD compared to HC. As scores for the impact of fatigue in the physical domain increase, functional connectivity between the **(A)** right Superior Parietal Lobule and left anterior parahippocampal gyrus, and **(B)** left Superior Parietal Lobule and left anterior parahippocampal gyrus decreases for the CD group, but not for the HC group.

The group contrasts comparing Crohn's disease to healthy control revealed significant differences in the association between FIS_Physical scores and the functional connectivity between the bilateral SPL and the left anterior PHG (TFCE = 16.88, *p*-FDR = 0.03). Unlike the healthy control group, an increasing impact of physical fatigue was associated with decreasing functional connectivity between these ROIs for the Crohn's disease group (Figure 2). The association between functional connectivity and scores on the FIS_Total and FIS_Cognitive did not differ between the Crohn's disease and healthy control groups.

### 3.3 Moderation analyses

Scores on the FIS_Total were positively correlated with HBI scores (*r* = 0.553, *p* < 0.001, *LLCI* = 0.312 *ULCI* = 0.726, *p* = 0.0002). Functional connectivity significantly moderated the relationship between HBI and FIS_Total for two out of the five ROI-pairings identified in the initial group-level analyses. Specifically, Model 1 revealed a significant moderating effect for functional connectivity between the right SPL and the left aPHG (ΔR^2^ = 0.058, *F* = 5.445, *p* = 0.0245). Greater disease activity was significantly associated with total impact of fatigue when functional connectivity between the right SPL and left aPHG was negative [i.e., when the signal timeseries for these regions was negatively correlated (β = 0.711, *SE* = 0.177, *t* = 4.022, *LLCI* = 0.354 *ULCI* = 1.068, *p* = 0.0002)]. FIS_Total scores were not significantly related to HBI scores when functional connectivity between these regions was either weakly positive (β = 0.313, *SE* = 0.164, *t* = 1.913, *LLCI* = −0.017 *ULCI* = 0.643, *p* = 0.063) or more strongly positive (β = −0.052, *SE* = 0.272, *t* = −0.190, *LLCI* = −0.599 *ULCI* = 0.496, *p* = 0.850). Similar, albeit weaker, findings were revealed for Model 3, showing a significant moderating effect for functional connectivity between the right SPL and the left Hipp (ΔR^2^ = 0.056, *F* = 4.060, *p* = 0.05). Greater total impact of fatigue was associated with greater levels of disease activity when functional connectivity between the right SPL and left Hipp was negative, at stronger (β = 0.901, *SE* = 0.209, *t* = 4.311, *LLCI* = 0.479 *ULCI* = 1.323, *p* = 0.0001) or weaker (β = 0.582, *SE* = 0.162, *t* = 3.595, *LLCI* = 0.255 *ULCI* = 0.909, *p* = 0.0008) negative correlations. FIS_Total scores were not significantly related to HBI scores when functional connectivity between these regions was positive (β = 0.068, *SE* = 0.322, *t* = 0.211, *LLCI* = −0.582 *ULCI* = 0.718, *p* = 0.834). Corresponding values for functional connectivity (i.e., Fisher-transformed correlation coefficients for signal time courses of specified ROI-pairings) are shown in Supplementary Figure 2. Models 2, 4, and 5 did not find a significant moderating effect for functional connectivity between the remaining ROI-pairs.

## 4 Discussion

The present study provides novel insight into brain-gut axis dysregulation in Crohn's disease and its role in the relationship between disease activity and the impact of fatigue. Expanding on prior studies that identified differences in brain structure associated with fatigue in Crohn's disease, the current study identified an inverse relationship between resting state functional connectivity and the total impact of fatigue, as well as its impact in both physical and cognitive domains for persons with Crohn's disease. Unlike healthy controls, who did not exhibit a significant association between fatigue and functional connectivity, an increasing impact of physical fatigue was associated with decreasing functional connectivity between the bilateral SPL and the left anterior PHG in the Crohn's disease group. Finally, the present findings revealed a moderating role for functional connectivity between the right SPL and the left anterior PHG/Hipp in the relationship between disease activity and total fatigue among persons with Crohn's disease. Collectively, this study identified previously unknown associations between the impact of fatigue and resting state functional connectivity in Crohn's disease and uncovered an interaction between disease activity, functional connectivity, and fatigue that may provide insight into the underlying pathophysiology of this common and debilitating symptom for persons with Crohn's disease.

The experience of increased fatigue in IBD is well established (Nocerino et al., 2019; Graff et al., 2011; D'Silva et al., 2022; Schreiner et al., 2020; Villoria et al., 2017). In line with the known burden of fatigue in IBD, our findings further validate the greater impact of fatigue for those with Crohn's disease compared to healthy adults. In line with abundant research on the prevalence and impact of fatigue among persons with IBD, our study is among the first to show how brain resting state functional connectivity relates to the self-reported impact of fatigue across separate domains of functioning. We found fatigue-related decreases in resting state functional connectivity of parieto-temporal regions in Crohn's disease. Fatigue-related resting state hypoconnectivity between the SPL and Hipp has also been reported in the context of chronic fatigue syndrome (Boissoneault et al., 2016). In addition, the parahippocampus has been shown to exhibit a negative relationship between cortical thickness and fatigue in people with Crohn's disease (Thapaliya et al., 2022). The SPL has a central role in somatosensory processing and sensorimotor integration (Passarelli et al., 2021), in addition to attention and memory processes (Wang et al., 2014; Fischer et al., 2020). Meanwhile, the Hipp and PHG are central components of the limbic system, critically involved in memory and affective processing (Catani et al., 2013). Altered functioning of the PHG and Hipp has been implicated in impaired self-awareness across several neurodegenerative diseases (Chavoix and Insausti, 2017). As such, diminished and anti-correlated synchronicity between brain regions that are involved in sensorimotor integration and memory processing/self-awareness is associated with the impact of fatigue in physical and cognitive functioning in Crohn's disease.

The impact of fatigue in the physical domain was also inversely related to functional connectivity between the right anterior SMG and the left pPHG in Crohn's disease. The SMG has been implicated in fatigue-related hypoconnectivity within the salience network in chronic fatigue syndrome (Zinn et al., 2016). Boissoneault et al. (2016) specifically reported a negative association between fatigue and functional connectivity of the left PHG and SMG. In what has been posited as an extension of its role in proprioception (Ben-Shabat et al., 2015), the right SMG has also been found to facilitate the ability to make self- vs. other-distinctions, reducing ego-centric biases and supporting emotion regulation (Silani et al., 2013). Hence, negatively related SMG functional connectivity may suggest that dysregulated proprioceptive processing, or possibly emotion dysregulation, could be associated with how persons with Crohn's disease experience the impact of fatigue in the physical domain. Indeed, other research has demonstrated an interaction between emotion processing, disease activity and fatigue in Crohn's disease such that higher disease activity is associated with greater disruption of emotional processing, leading to increased fatigue (Banovic et al., 2020). The present findings may therefore suggest a potential therapeutic target for interventions aimed at improving emotion processing (contributing to reduced fatigue) by increasing functional connectivity between the SMG and pPHG.

Notably, no significant associations were observed for the impact of fatigue in the psychosocial domain of functioning, nor were any significant associations between the impact of fatigue and functional connectivity observed for the healthy control group. Compared to the physical and cognitive impacts of fatigue in varied clinical populations, the impact of fatigue in the psychosocial domain has been less frequently studied (Billones et al., 2021). Increased physical and cognitive impacts of fatigue for person with Crohn's disease have been reported compared to both ulcerative colitis and healthy controls (van Langenberg and Gibson, 2014). Despite significant between-group differences across each domain of fatigue measured by the FIS in the current data, scores not indicate extreme fatigue in any domain. Future research including participants with more extreme fatigue may elucidate neural correlates for the impact of fatigue in the psychosocial domain. However, impacts of fatigue on functions within a social context may be so diverse and context-dependent between individuals that clear associations with brain functional connectivity differences may be challenging to identify. Lack of findings for a significant association between the impact of fatigue and functional connectivity for the healthy control group was unsurprising given the low range and limited variability in FIS scores within that group. A greater range in FIS scores in the control group could strengthen analyses aimed at detecting significant associations with resting state functional connectivity (Seghier and Price, 2018). Among the FIS subscales, scores in the physical domain showed the greatest difference for Crohn's disease compared to healthy controls. The difference between the Crohn's disease and the healthy control group in the association between functional connectivity and physical fatigue scores is in line with the largest difference in self-reported impact of fatigue in that domain. It is possible that future research including participants with more extreme fatigue would reveal further differences among the other subscales or relative to total scores on the FIS in direct comparison between persons with Crohn's disease and healthy controls.

Beyond revealing that functional connectivity of the identified parieto-temporal regions decreases as the impact of fatigue increases in people with Crohn's disease, the present findings also identify an interaction between disease activity, functional connectivity, and the impact of fatigue. The positive association between disease activity and the impact of fatigue is only observed for participants who exhibit negative resting state functional connectivity between the right SPL and the left aPHG, as well as the left Hipp. Among persons with Crohn's disease who exhibit positive functional connectivity between these regions, the association between disease activity and fatigue is not significant. Given that fatigue in IBD has been shown to increase over time even when disease state has gone into remission (Graff et al., 2013), identifying potential moderating factors of the relationship between disease activity and fatigue provides a critical advancement to understanding the etiology of fatigue in Crohn's disease. These results highlight resting state functional connectivity between these regions as a characteristic of brain-gut axis dysregulation in Crohn's disease, linking specific changes in brain function to the relationship between disease activity and fatigue. Future research may explore whether interventions aimed at increasing functional connectivity between the identified parieto-temporal regions [e.g., through the use of real time fMRI-feedback (Kim et al., 2019; Mehler et al., 2018)] may uncouple fatigue and disease activity in IBD.

### 4.1 Limitations and future directions

Depression is typically prevalent among those with IBD (Walker et al., 2008; Kredentser et al., 2020), and is strongly linked to fatigue (Uhlir et al., 2023; Bernstein et al., 2023). For the present study, diagnosis of any mental health disorder was an exclusion criterion for participation among healthy controls but not among those with Crohn's disease, so the group-difference on HADS-D scores was unsurprising. Regardless, our participant sample was unconfounded by clinically meaningful depression in that neither group had significantly elevated mean scores (≥11) on the HADS-D; only one participant with Crohn's disease (and none of the healthy controls) had a score ≥11. Scores from the HADS-D were, therefore, not included as a control covariate in the functional connectivity analyses. Similarly, disease duration was not considered in the present analyses given the null effect on resting state functional connectivity reported in previous research (Kornelsen et al., 2020). With so few studies having examined neural correlates of fatigue in IBD, the novel insight provided by the present study should facilitate a more comprehensive approach in future research aimed at distinguishing neural correlates of fatigue in participants that exhibit clinical levels of depressive symptoms and other related comorbidities. Further, while we report that medications were used in the form of immunosuppressants and biologics among 34 of the participants with Crohn's Disease, the current study did not statistically control for a potential influence of medication use in the current analyses. Future studies may aim to explore whether use of immunosuppressants or biologics differently influence fatigue-related resting state functional connectivity among persons with Crohn's disease.

Future research should explore the causal mechanisms/directionality of the identified interaction between disease activity, functional connectivity, and fatigue. At present, it is unclear whether the reported functional connectivity patterns precede or follow rises in disease activity. Without knowing the temporal order of these changes, our analysis of the interaction between disease activity, functional connectivity, and fatigue was limited to exploring a moderating (rather than potential mediating) role of functional connectivity. Longitudinal research designs are sorely lacking in resting state fMRI research in IBD and would help inform more refined *a priori* hypotheses about the role of functional connectivity in the relationship between disease activity and fatigue. Further, in line with Thomann et al. (2024), a multi-modal approach incorporating additional biomarkers of inflammation such as fecal calprotectin would provide a more comprehensive understanding of how various risk factors interact to influence the impact of fatigue in IBD. In addition, future application of large-scale data can be used to build predictive models based on artificial intelligence to predict patient outcome as well as identifying markers for early diagnosis.

### 4.2 Conclusion

While fatigue-related brain structure differences have previously been reported in persons with IBD, the present study is the first to our knowledge, to find a negative relationship between the impact of fatigue and resting state functional connectivity among those with Crohn's disease, as well as the first to reveal a significant interaction between disease activity, functional connectivity, and the impact of fatigue in Crohn's disease. Functional connectivity differences between persons with Crohn's disease and healthy controls emerged in the context of the physical impact of fatigue, highlighting that different aspects of fatigue may be related to distinct neural correlates. The negative association between the impact of fatigue and functional connectivity between brain regions involved in processing sensorimotor integration, motor planning, and memory may implicate weakened coordination of these processes as a potential factor contributing to the experience of fatigue among those with Crohn's disease.

## Data Availability

The datasets presented in this article are not readily available because the authors do not have permission to share data collected for this study. Requests to access the datasets should be directed to Jennifer Kornelsen, jennifer.kornelsen@umanitoba.ca.
