# Supplementary material for: Impact of fatigue in Crohn's disease is negatively related to resting state functional connectivity between the superior parietal lobule and parahippocampal gyrus/hippocampus
Source: Front Hum Neurosci. 2025 Apr 25;19:1561421. doi: 10.3389/fnhum.2025.1561421 (PMC12062172; doi:10.3389/fnhum.2025.1561421)
Supplement: Supplementary file 1 [file Data_Sheet_1.docx]

Supplementary Material

# Supplementary Methods

## Participant Recruitment

Inclusion criteria for all study participants included being ≥ 18 years of age, English-speaking, and screening clear of MRI contraindications, history of brain tumor, neurodegenerative disease, or immune-mediated inflammatory disease (other than Crohn’s disease for the patient group). Healthy controls were recruited via posters placed in hospital, university, and community settings in Winnipeg, direct mail, and word of mouth. In addition to the exclusion criteria for participants with Crohn’s disease recruitment, healthy controls were additionally screened for history of traumatic brain injury with loss of consciousness or amnesia, chronic medical conditions including hypertension, intellectual disability or cognitive impairment, psychiatric disorders on the Structured Clinical Interview for DSM-IV, and any chronic medication use other than contraceptives or hormone replacement therapy, as detailed previously(1) (2).

## Scanning Parameters

High resolution anatomical, whole-brain T1-weighted (T1w) images were acquired using a 3D magnetization prepared rapid acquisition gradient-echo (MPRAGE) sequence with the following scan parameters: repetition time [TR] = 1,900 ms, echo time [TE] = 2.47 ms, inversion time [TI] = 900 ms, flip angle = 9◦, GRAPPA = 2, matrix size = 256 × 256, field of view [FOV] = 250 × 250 mm^2^, number of slices = 176, slice thickness = 1.00 mm, number of averages = 1, bandwidth [BW] = 170 Hz/Px, echo spacing [ESP] = 7.3 ms, spatial resolution = 0.98 × 0.98 × 1.00 mm^3^, acquisition time [TA] = 4:26 min.

Resting state fMRI data were acquired using a gradient-echo MB-EPI sequence with the following imaging parameters: TR = 1,000 ms, TE = 38.6 ms, flip angle = 61◦, matrix size = 100 × 78; FOV = 220 × 171.6 mm^2^, number of slices = 80, slice thickness = 2.20 mm, MB factor = 8, number of volumes = 420, BW = 2,272 Hz/Px, ESP = 0.78 ms, EPI factor = 78, spatial resolution = 2.20 × 2.20 × 2.20 mm^3^, TA = 7:12 min.

## Neuroimaging Preprocessing

The first 10 scans in each functional run were removed. Functional data were realigned using SPM realign & unwarp procedure(3), where all scans were coregistered to a reference image (first scan of the first session) using a least squares approach and a 6 parameter (rigid body) transformation(4), and resampled using b-spline interpolation to correct for motion and magnetic susceptibility interactions. Potential outlier scans were identified using ART(5) as acquisitions with framewise displacement above 0.9 mm or global BOLD signal changes above 5 standard deviations(6), and a reference BOLD image was computed for each subject by averaging all scans excluding outliers. Functional and anatomical data were normalized into standard MNI space, segmented into grey matter, white matter, and CSF tissue classes, and resampled to 2 mm isotropic voxels following a direct normalization procedure(7) using SPM unified segmentation and normalization algorithm(8,9) with the default IXI-549 tissue probability map template. Last, functional data were smoothed using spatial convolution with a Gaussian kernel of 8 mm full width half maximum (FWHM). In addition, functional data were denoised using a standard denoising pipeline(10) including the regression of potential confounding effects characterized by white matter timeseries (5 CompCor noise components), CSF timeseries (5 CompCor noise components), motion parameters and their first order derivatives (12 factors)(11), outlier scans (below 82 factors)(6), session effects and their first order derivatives (2 factors), and linear trends (2 factors) within each functional run, followed by bandpass frequency filtering of the BOLD timeseries(12) between 0.008 Hz and 0.09 Hz. CompCor(13,14) noise components within white matter and CSF were estimated by computing the average BOLD signal as well as the largest principal components orthogonal to the BOLD average, motion parameters, and outlier scans within each subject's eroded segmentation masks.

**2 Results**

**2. 1 Medication Use**

Medication use among participants with Crohn’s disease included 5-ASA|5-ASA oral (n=1|5), thiopurines (n=13), methotrexate (n=2), antibodies to TNF (n=19), Ustekinumab (n=6), vedolizumab (n=1). Fifteen participants with Crohn’s disease were not using any IBD medication.

**2.2 Supplementary Figures & Figure Captions**

**Supplementary Figure 1.** **Scatterplots displaying the negative association between FIS_Physical scores and functional connectivity for the CD group.** Functional connectivity from all of the same ROI-pairings were found to be significantly negatively related to the FIS_Cognitive and FIS_Total (i.e. as the impact of fatigue increases, functional connectivity between these ROI-pairings decreases, from positive to negative connectivity), except the right anterior Supramarginal Gyrus – left posterior Parahippocampal gyrus (red box outline).

Alt text: Six scatterplots are displayed, one for each of the ROI-pairs between which functional connectivity was found to be significantly, negative correlated with FIS_Physical scores. Each graph shows fatigue scores plotted on the x-axis and functional connectivity values plotted on the y-axis, with a line of best fit displayed through the individual data points.

**Supplementary Figure 2.**

**Visualization of the moderating role for resting state functional connectivity on the positive association between disease activity (HBI scores) and total impact of fatigue (FIS_Total).** A) Model representing the hypothesized moderation of the relationship between disease activity and the impact of fatigue by resting state functional connectivity. B) Graph displaying the correlation between standardized scores for HBI and FIS_Total at low, medium, and high levels of functional connectivity between the right SPL – left aPHG (represented by Fisher-transformed correlation coefficients). The low level represents the 16th percentile (where functional connectivity was negative at *r* = -0.149); the medium level represents the 50th percentile (functional connectivity was near zero at *r* = +0.082); and the high level represents the 84th percentile (functional connectivity was stronger positive at *r* = +0.294). C) Graph displaying the correlation between standardized scores for HBI and FIS_Total at low, medium, and high levels of functional connectivity between the right SPL – left Hipp The low level represents the 16th percentile (where functional connectivity was negative at *r* = -0.280); the medium level represents the 50th percentile (functional connectivity was near zero at *r* = -0.069); and the high level represents the 84th percentile (functional connectivity was stronger positive at *r* = 0.2714).

Alt Text: A) A theoretical model for the moderating effect of resting state functional connectivity on the relationship between disease activity and fatigue is shown at the top of the multi-panel figure. B) Three lines of varying slopes are displayed, representing the relationship between HBI scores and FIS_Total scores at varying values for functional connectivity between the right SPL – left aPHG. C) Three lines of varying slopes are displayed, representing the relationship between HBI scores and FIS_Total scores at varying values for functional connectivity between the right SPL – left Hipp.

**Supplementary Figure 1.**


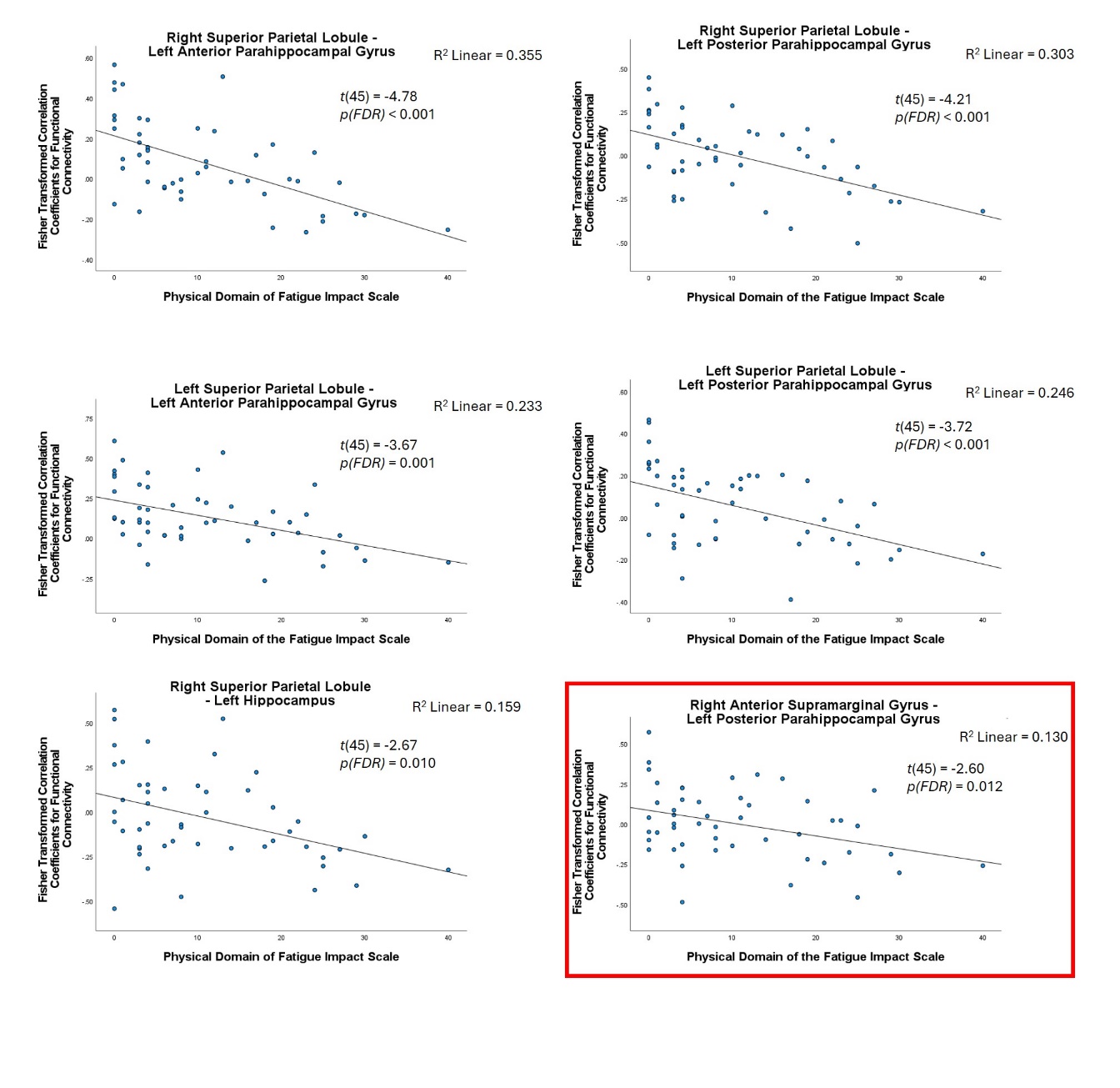


**Supplementary Figure 2.**

**
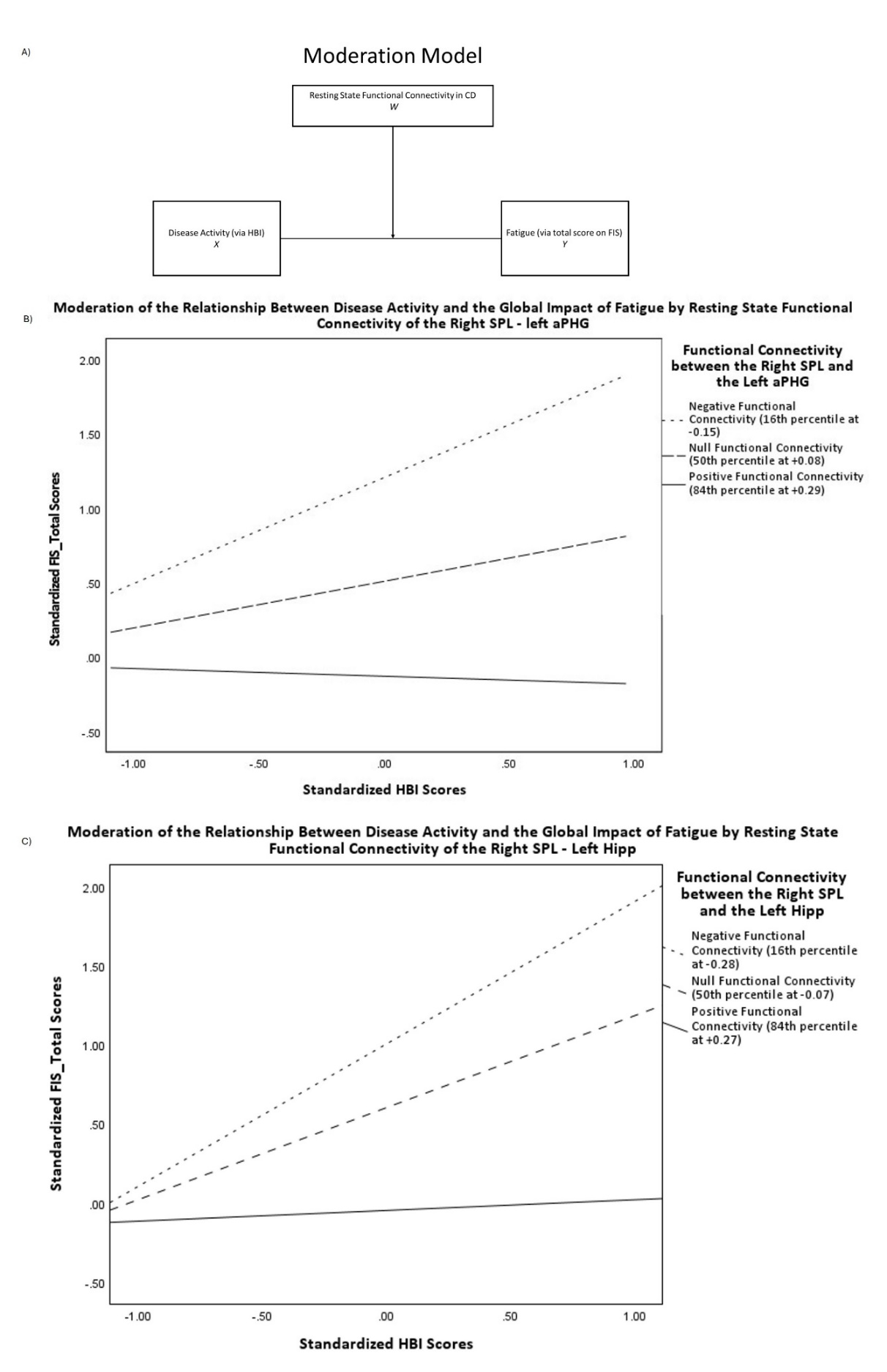
**

**References**

1. Marrie RA, Graff L, Walker JR, Fisk JD, Patten SB, Hitchon CA, et al. Effects of Psychiatric Comorbidity in Immune-Mediated Inflammatory Disease: Protocol for a Prospective Study. JMIR Res Protoc. 2018 Jan;7(1):e15.

2. Uddin MN, Figley TD, Kornelsen J, Mazerolle EL, Helmick CA, O’Grady CB, et al. The comorbidity and cognition in multiple sclerosis (CCOMS) neuroimaging protocol: Study rationale, MRI acquisition, and minimal image processing pipelines. Frontiers in Neuroimaging. 2022 Aug 24;1.

3. Andersson JLR, Hutton C, Ashburner J, Turner R, Friston K. Modeling geometric deformations in EPI time series. Neuroimage. 2001;13(5):903–19.

4. Friston KJ, Ashburner J, Frith CD, Poline JB, Heather JD, Frackowiak RSJ. Spatial Registration and Normalization of Images. Human Brain Mapping. 1995.

5. Whitfield-Gabrieli S, Nieto-Castanon A, Ghosh S. Artifact detection tools (ART). Release Version 7 (19), 11. Cambridge, MA; 2011.

6. Power JD, Mitra A, Laumann TO, Snyder AZ, Schlaggar BL, Petersen SE. Methods to detect, characterize, and remove motion artifact in resting state fMRI. Neuroimage. 2014 Jan 1;84:320–41.

7. Calhoun VD, Wager TD, Krishnan A, Rosch KS, Seymour KE, Nebel MB, et al. The impact of T1 versus EPI spatial normalization templates for fMRI data analyses. Hum Brain Mapp. 2017 Nov 1;38(11):5331–42.

8. Ashburner J, Friston KJ. Unified segmentation. Neuroimage. 2005 Jul 1;26(3):839–51.

9. Ashburner J. A fast diffeomorphic image registration algorithm. Neuroimage. 2007 Oct 15;38(1):95–113.

10. Nieto-Castanon A. FMRI denoising pipeline. In: Handbook of functional connectivity Magnetic Resonance Imaging methods in CONN. Hilbert Press; 2020. p. 17–25.

11. Friston KJ, Williams S, Howard R, Frackowiak RSJ, Turner R. Movement-related effects in fMRI time-series. Magn Reson Med. 1996;35(3):346–55.

12. Hallquist MN, Hwang K, Luna B. The nuisance of nuisance regression: Spectral misspecification in a common approach to resting-state fMRI preprocessing reintroduces noise and obscures functional connectivity. Neuroimage. 2013 Nov 5;82:208–25.

13. Behzadi Y, Restom K, Liau J, Liu TT. A component based noise correction method (CompCor) for BOLD and perfusion based fMRI. Neuroimage. 2007 Aug 1;37(1):90–101.

14. Chai XJ, Castañán AN, Öngür D, Whitfield-Gabrieli S. Anticorrelations in resting state networks without global signal regression. Neuroimage. 2012 Jan 16;59(2):1420–8.

**
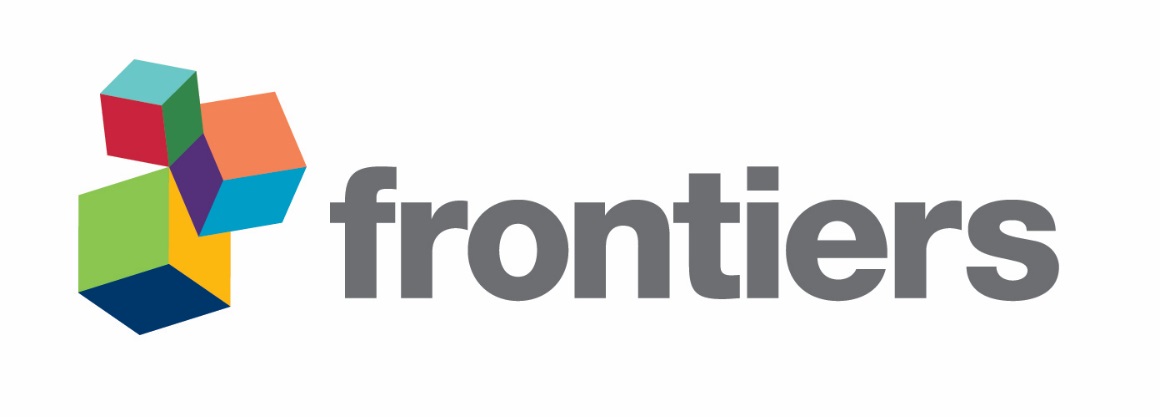
**
